# Accelerated Growth following Poor Early Nutrition Impairs Later Learning

**DOI:** 10.1371/journal.pbio.0040270

**Published:** 2006-07-18

**Authors:** Liza Gross

The conditions an organism experiences early in life can have critical impacts on its subsequent health and well being, both over the short and long term. Aside from facing a greater risk of death, low birth weight human babies (5.5 pounds and under) have increased risk of developmental disabilities throughout life. Recent evidence indicates that many organisms can offset some of the changes associated with early poor nutrition by modifying their physical development. For example, poorly nourished children can undergo a period of accelerated growth once their diet improves, ultimately appearing normal as an adult.

But such compensatory measures often come at a price, with cognitive or other developmental disabilities emerging later in life, suggesting that growth rates are optimized to avoid such costs. Poor nutrition early in life can impair neural development, leading to lower IQ in humans and flawed song learning in birds. A recent study found that full-term, low birth weight babies who grew quickly when fed an enriched diet had lower cognitive skills when tested at nine months than did babies given a normal diet. But questions remain about the relative consequences of compensatory growth versus impaired growth and poor nutrition on the observed cognitive defects.

In a new study, Michael Fisher, Rudolph Nager, and Pat Monaghan explored the connection between early poor nutrition, compensatory growth, and learning ability in adulthood. To circumvent the confounding variables inherent in human studies and to control for genetic effects, the researchers compared the learning performance of zebra finch siblings reared on different quality diets after hatching. Only food quality, not quantity, was changed. The rate at which adult birds could learn a simple task, they found, depended on the rate of compensatory growth the birds showed following a period on lower-quality food early in life—not on the diet itself or on the degree of stunted growth.

Because zebra finch hatchlings are totally dependent on their parents for food, the researchers could vary food quality for the chicks by manipulating the food available. The birds, facile learners that are often used in cognitive studies, were reared on ad libitum diets of different nutritional quality and then tested for learning performance as adults. (Handily, zebra finches show little sex differences in size as adults.) After hatching, siblings were raised on either a normal or low-quality diet for 20 days, then switched to the higher-quality standard diet. While on the low-quality diet, birds grew slower and were lighter than their control siblings by the end of the 20 days. Once they were switched to the standard diet, birds reared on the poor diet then grew significantly more than their normally fed siblings and reached the same adult size.

The extent to which birds’ growth was depressed during the poor nutrition phase of the experiment varied considerably, as did the degree of accelerated growth after the switch to a normal diet. As it happened, birds with the most stunted growth (relative to their control siblings) and those with the most accelerated growth (after switching diets) fell into different groups, allowing the researchers to distinguish cognitive effects associated with stunted growth from those associated with compensatory growth.

To test the adult birds’ learning performance, the researchers tested them on an associative learning task. Birds were placed in a circular foraging area with corridors leading to a screen with cups of seed behind it, and were trained to associate a yellow screen with food. Though all the birds eventually learned the task, their learning rate depended on the rate of compensatory growth they had undergone as chicks. Undernourished birds that had grown fastest after switching to the normal diet performed poorest on the learning task compared to their control siblings. Since the undernourished birds were the only group that showed this relationship between growth rate and learning speed, the researchers concluded that it is the compensatory growth following reduced nutrition that accounts for poor learning performance in adulthood.

These results suggest that poor early nutrition can have long-lasting negative consequences for cognitive ability—for finches as well as humans, given similar findings in human infants. While it’s unclear whether the learning defects stem from behavioral, hormonal, or neural changes, it’s likely that resources normally dedicated to these pathways are diverted to support accelerated growth, shortchanging the co-opted pathway. Future study is needed to identify the underlying causes of impaired learning speed, an essential step in determining how to manage growth and nutrition for low birth weight babies and avoid the costs associated with compensatory growth.

## 

**Figure:**
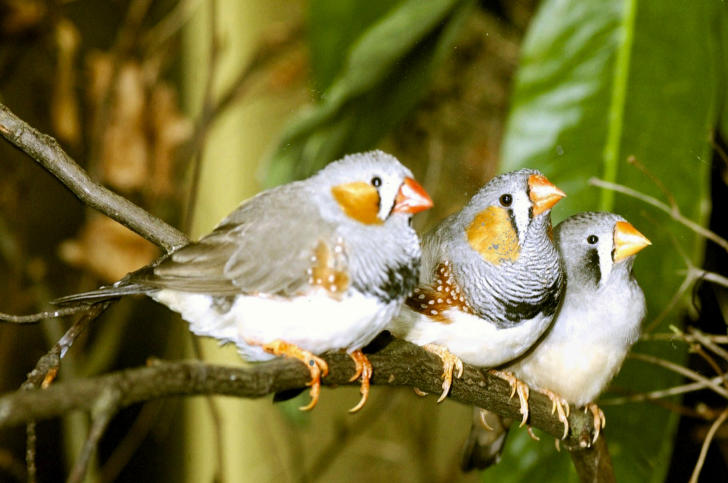
Adult zebra finches who grew fastest as chicks after a nutrition-poor diet had trouble learning an association task.

